# MicroRNA-320d regulates tumor growth and invasion by promoting FoxM1 and predicts poor outcome in gastric cardiac adenocarcinoma

**DOI:** 10.1186/s13578-020-00439-7

**Published:** 2020-06-16

**Authors:** Xiaojie Chen, Shegan Gao, Zhiwei Zhao, Gaofeng Liang, Jinyu Kong, Xiaoshan Feng

**Affiliations:** 1 Medical College, Henan University of Science and Technology, Luoyang, China; 2 The First Affiliated Hospital, College of Clinical Medicine of Henan University of Science and Technology, No. 263, Kaiyuan Street, Luolong District, Luoyang, 471000 China; 3 Henan Key Laboratory of Cancer Epigenetics, Henan University of Science and Technology, Luoyang, China; 4 Cancer Institute, Henan University of Science and Technology, Luoyang, China; 5 China-US (Henan) Hormel Cancer Center, Zhengzhou, Henan 450008 China

**Keywords:** MicroRNA-320d, FoxM1, Gastric cardiac adenocarcinoma, Prognosis, Proliferation, Migration

## Abstract

Recent evidences demonstrate that dysregulated expression of microRNA-320d (miR-320d) has been associated with several cancer development and progression. However the effect of miR-320d on gastric cardiac adenocarcinoma (GCA) and the association of miR-320d with its potential gene target FoxM1 remain unclear. Here, we evaluated expression profile of miR-320d and FoxM1 in 60 human GCA tissues and GCA cell lines (OE-19 and SK-GT2). Immunohistochemistry, qualitative PCR and western-blotting were performed in GCA tissues to detect the expression level of miR-320d and FoxM1. CCK-8, transwell, wound-healing assays, and in vivo experiments were conducted using GCA cells that treated with miR-320d mimics or inhibitors to evaluate the biological functions of miR-320d. Luciferase reporter assay was conducted to confirm possible binding sites of FoxM1 for miR-320d. Compared with paired non-cancerous tissues, it showed that miR-320d expression was significantly decreased in GCA specimens (*P* < 0.0001), while FoxM1 was significantly upregulated in GCA tissues (*P* < 0.0001). Modulating miR-320d function by transfection of miR-320 mimics or inhibitor led to inhibition or promotion of GCA cell proliferation and invasion, thus regulating tumor progression in GCA-tumor bearing mice. The mechanism analysis of miR-320d/FoxM1 showed that FoxM1 has two miR-320d binding sites in its 3′-untranslated region (3′-UTR), that contributes to regulation of the cell biological behaviors. Taken together, our data suggested that miR-320d acts as a tumor suppressor in GCA by directly targeting FoxM1 and thus potentially serves as a biomarker for anti-GCA therapy in GCA patients.

## Introduction

According to nationwide statistics of cancer incidence and mortality in China, gastric cancer ranks as the second prevalent cancer type, and the third most common cause of death [1]. Unlike other types of gastric cancer, gastric cardia adenocarcinoma (GCA) is a special aggressive cancer and has its own epidemiological and pathological characteristics [2, 3]. Over the recent decades, the incidence of GCA has dramatically increased, especially in the United States and Northern China [4–7]. Accumulated studies have demonstrated that the dysregulation of tumor suppressors [3, 8–10] and oncogenes [11, 12] was associated with carcinogenesis, metastasis, and poor prognosis of GCA. However, the molecular mechanisms behind GCA still remained unclear.

The microRNAs (miRNAs) are small noncoding RNAs with 20-to-22 nucleotides which can regulate protein translation through mediating either mRNA degradation or translational repression [3, 13], and thus play a crucial role throughout the physiological processes of cells, including development, metabolism, proliferation, differentiation, and apoptosis [14, 15]. However, the dysregulation of miRNAs can also contribute to carcinogenesis by regulating oncogenes at the post-transcriptional level [3]. Until now, more than 1000 miRNAs are reported in encoding in the human genome. Among these revealed miRNAs, microRNA-320d (miR-320d), a member of miR-320 family, has been reported to be involved in different malignant tumors, including large B cell lymphoma [14], colon cancer [15], renal cell carcinoma [16]. However, studies focusing on the role and mechanism of miR-320d in the progression and development of GCA are still lacking.

In this study, we verified the prognosis value of miR-320d in human GCA tissue, and identified its expression level in GCA specimens, GCA cell lines, and GCA tumor-bearing mice. The miR-320d function in GCA cell line was modulated by either transfection with miR-320d mimics or inhibitor. Cell proliferation, migration, and invasion assays were performed to evaluate the effects of miR-320d on GCA cells after transfection in vitro. Meanwhile subcutaneous GCA mouse model was developed to explore the effect of miR-320d in vivo. Through TargetScan database, we found that there were two binding sites of a nuclear protein, Forkhead box M1 (FoxM1), for miR-320d targeting. As to reveal the relationship between FoxM1 and miR-320d, we evaluated the FoxM1 expression either after inhibiting or enhancing of miR-320d function both in vitro and in vivo. Two binding sites between FoxM1 and miR-320d were revealed by dual luciferase assay.

## Materials and methods

### Patient specimens

This study was approved by the Ethics Committee of the Henan University of Science and Technology and in accordance with the declaration of Helsinki and carried out with informed consent from all patients. A total of 60 specimens and paired non-cancerous specimens were gained from GCA patients at the First Affiliated Hospital of Henan University of Science and Technology or Cancer hospital in Anyang city between April 2014 and June 2016. All patients pathologically confirmed with GCA and without receiving radiotherapy or chemotherapy before surgery were included. The detailed patient characteristics were summarized in Table 1.

**Table 1:** Clinicopathological features of 60 patients with GCA

Characteristics	Grouping	No. of patients	Percentage (%)
Gender	Male	44	73.3
	Female	16	26.7
Age (years)	< 60	19	31.7
	≥ 60	41	68.3
The stomach diseases	No	18	30.0
	Yes	42	70.0
Family history of cancer	No	33	55.0
	Yes	27	45.0
Tumor size (cm)	< 5	27	45.0
	≥ 5	33	55.0
Pathological grade	Low differentiated	19	31.7
	Middle and high differentiated	41	68.3
Clinical stage	Low grades I–II	12	20.0
	High grades III–IV	48	80.0
Lymph node metastasis	No	10	16.7
	Yes	50	83.3
Follow-up status	Survival	44	73.3
	Death	14	23.3
	Loss to follow-up	2	3.3
Total		60	100.0

*GCA* Gastric cardia adenocarcinoma

### Immunohistochemistry (IHC) analysis

The IHC analysis was performed in accordance with the standard protocol. Briefly, the paraffin-embedded tissue specimens were sectioned and incubated with anti-FoxM1 primary antibody (1:200, Abcam, USA) overnight at 4 °C. The tissues treated with PBS were set as controls. Then biotinylated secondary antibody (goat anti-rabbit IgG, BOSTER, China) and peroxidase-coupled streptavidin (BOSTER, China) were successively applied on the slices and incubated at room temperature. After that, DAB (3.3′-diaminobenzidine tetrahydrochloride, BOSTER, China) was used as chromogen for positive FoxM1 visualization. The resulting slices were analyzed by two independent pathologists, and the IHC results were measured as previously described [17–21].

### Cell culture

GCA cell lines (OE-19 and SK-GT2) were gifted from Dr. Wang Huizhi (Microbes and the immune laboratory in the University of Louisville, USA). Cells were cultured in RPMI-1640 medium (Gibco™, USA) with 10% fetal bovine serum (FBS, Biological Industries, Israel), and 1% Penicillin–Streptomycin Solution (Solarbio, Beijing) at 37 °C in a 5% CO_2_ incubator.

### Cell transfection

The human miR-320d mimics, miR-320d inhibitor, and empty vector (EV) plasmid were purchased from GeneCopeia™ Company (USA). SK-GT2 cells (high expression level of miR-320d) were transfected with miR-320d inhibitor (*Lenti*-*Pac*™ Lentiviral Packaging), and OE-19 cells (low expression level of miR-320d) was transfected with miR-320d mimics, following the manufacturer’s instructions. For subsequent luciferase assays, OE-19 cells were transfected either with FoxM1 promoter (pEZX-LvGA04-FoxM1, GeneCopeia™, USA) or EV plasmid (control group).

### Reverse transcription-quantitative polymerase chain reaction (RT-qPCR)

The total RNA was extracted from GCA cells before or after transfection, by using Trizol Reagent (Invitrogen, USA). The reverse transcription was performed using the cDNA Synthesis Kit (Vazyme, USA) according to the manufacture’s protocol. The commercialization primers for miR-320d and U6 were purchased from GeneCopeia™, and the sequence of primers for FoxM1 and GAPDH (synthetized by GeneCopeia™ company) was shown in Additional file 1: Table S1. U6 or GAPDH was used as normalizing control. PCR was performed on RT-qPCR Super Mix (Vazyme, USA). Relative RNA expressions were calculated by the 2^−ΔΔCT^ method.

### Western blotting

The fresh frozen tissues from GCA patients or cultured GCA cells were lysed with RIPA lysis buffer (Sigma, USA). A total of 40 µg protein was loaded and separated by SDS-PAGE, and transferred to NC membranes (Millipore, Germany). The total proteins were incubated with FoxM1 primary antibody (1:1000, Abcam, USA) and GAPDH antibody (Cell signaling, USA) at 4 °C for overnight. Then horseradish peroxidase (HRP)-conjugated secondary antibody (goat anti-rabbit IgG, 1:3000, BOSTER, China) was added and incubated at room temperature for 2 h. The immunocomplex on the membrane was visualized using an ECL kit (Biovision, USA). The images were scanned by BIO-RAD CHEMIDOC XRS + imaging system and analyzed by Image Lab™ imaging software (BIO-RAD, USA).

### Cell proliferation assays

OE-19, miR-320d mimics-transfected OE-19, SK-GT2, miR-320d inhibitor-transfected SK-GT2 cells were seeded in 96-well plates at the density of 2000 cells per well, and incubated for 24 h, 48 h, and 72 h. At the endpoint, 20 µL CCK-8 reagent (Dojindo, Japan) in 100 µL culture medium was added into the plates according to the manufacturer’s protocol, followed by incubation at 37 °C for 4 h. Then 100 µL supernatant in each well was collected and absorbance at wavelength of 450 nm was measured on an automatic microplate reader (Enspire™ Company). The experiment was performed in triplicate.

### Colony formation assays

OE-19, miR-320d mimics-transfected OE-19, SK-GT2, or miR-320d inhibitor-transfected SK-GT2 cells were seeded in 6-well plates at the density of 200 cells per well, and cultured for up to 2 weeks. The cell colonies were stained with 0.1% crystal violet (HBK Pharmaceutical Technology Co., China) for 10 min. After rinsed with PBS, the colonies with > 50 cells were counted. The experiment was performed in triplicate.

### Migration and invasion assay

Cell migration activity was determined by using 8-μm transwell inserts (Millipore, USA). EV or miR-320d mimics transfected OE-19 cells in 200 µL serum-free culture medium were seeded into the upper chambers of inserts, and the lower compartment was filled with 500 µL medium containing 25% FBS. After a migration time of 24 h at 37 °C, non-migrating cells on the upper membrane of inserts were removed by wiping with a cotton swab, and the cells adhering to the bottom side of inserts were fixed with ethyl alcohol for 10 min, followed by staining with crystal violet for 10 min. After rinsed with PBS, migrating cells were imaged by microscope, and manually counted.

Cell invasion activity was determined by using wound-healing assay. EV or miR-320d mimics transfected OE-19 cells, or EV or miR-320d inhibitor-transfected SK-GT2 cells were seeded in 6-well plates at the density of 4 × 10^5^ cells per well. After achieving 80% cell confluence, a straight line was made using a 200-µL pipette tip. The remaining cells were incubated for additional 24 h in fresh culture medium. Bright field images along the scrape line were taken, and the line width was measured at 0 h and 24 h, respectively.

### Dual luciferase reporter assay

The plasmid containing FoxM1 3′-UTR for the putative miR-320d binding and luciferase reporter gene was constructed by using pEZX vector (GeneCopeia™). Two mutants of FoxM1 3′-UTR were constructed by the GeneCopeia™ company. The oligonucleotide sequences for FoxM1 3′-UTR mutated types and wild type were listed in Additional file 1: Table S2. Then OE-19 cells were transfected with constructed plasmids by lipofectamine™ 2000 (Invitrogen, USA), and group settings were shown as followed (Additional file 1: Table S3, n = 5 for each group): (1) negative control, cells transfected with NC plasmid and wild FoxM1 3′-UTR plasmid; (2) positive control, cells transfected with stable expression of miR-320d plasmid and wild type FoxM1 3oxM1 plasmid; (3) cells transfected with stable expression of miR-320d plasmid and mutant site 1 of FoxM1 3′-UTR plasmid; (4) cells transfected with stable expression of miR-320d plasmid and mutant site 2 of FoxM1 3′-UTR plasmid; (5) cells transfected with stable expression of miR-320d plasmid and mutant site 1 and 2 of FoxM1 3′-UTR plasmid. After the transfection, cells were harvested and analyzed for luciferase activity using Luc-Pair ™ Duo-Luciferase Assay Kit 2.0 (GeneCopeia™).

### Animal experiments

Five**-**week old male athymic nude mice were purchased from the Experimental Animal Center Xuzhou Medical College and maintained in the Experimental Animal Center at Medical College, Henan University of Science and Technology under specific pathogen-free conditions. All the animal experiments reported here were carried out in accordance with the approved guideline and approved by the committee on the Animal Care and Use of Medical College, Henan University of Science and Technology.

OE-19, miR-320d mimics-transfected OE-19, SK-GT2, or miR-320d inhibitor-transfected SK-GT2 cells (1 × 10^6^ cells) were subcutaneously transplanted into the right flanks of the nude mice (n = 5 per group). Tumor size measurement was initiated when the tumor reached 100–150 mm^3^. Tumor volume was calculated according to the formula: V = (a × b^2^)/2, where a and b are the maximal and minimal diameter of tumors, respectively. After 4 weeks for tumor growth, the mice were sacrificed by cervical dislocation. Tumors were harvested, weighted, and preserved in − 80 °C for further analysis. The detection of FoxM1 miRNA level and protein level in each tumor sample was performed following the descriptions in the RT-qPCR and western blot sections. The histological analysis of FoxM1 in tumor tissue was described as before.

### Statistical analysis

The SPSS statistical software package (SPSS, version 17.0, Chicago, IL, USA) was used in this study for statistical evaluation. All data were carried out at least in triplicate, and presented as the mean ± SD. The one-way analysis of variance (ANOVA) and two-way ANOVA were performed to assess statistical significance of the results (*: P < 0.05, **: P < 0.01, and ***: P < 0.001). The correlation between miR-320d and FoxM1 mRNA expression was analyzed using the Spearman’s rank correlation. Kaplan–Meier method and log-rank test were used for human patient survival study.

## Results

### MiR-320d is negatively correlated with FoxM1 in GCA tissues, both are associated with prognosis of GCA patients

Firstly, we aimed to determine the miR-320d’s target genes and the binding sites. Based on the available online bioinformatics websites, we found that there are two potential binding sites between the FoxM1 3′-UTR and miR-320d, and the context score of these sites are high, which suggested FoxM1 may serve as a potential target gene for miR-320d. Therefore, we explored miR-320d/FoxM1 correlation in the following in vitro, ex vivo, and in vivo experiments.

**Fig. 1:**
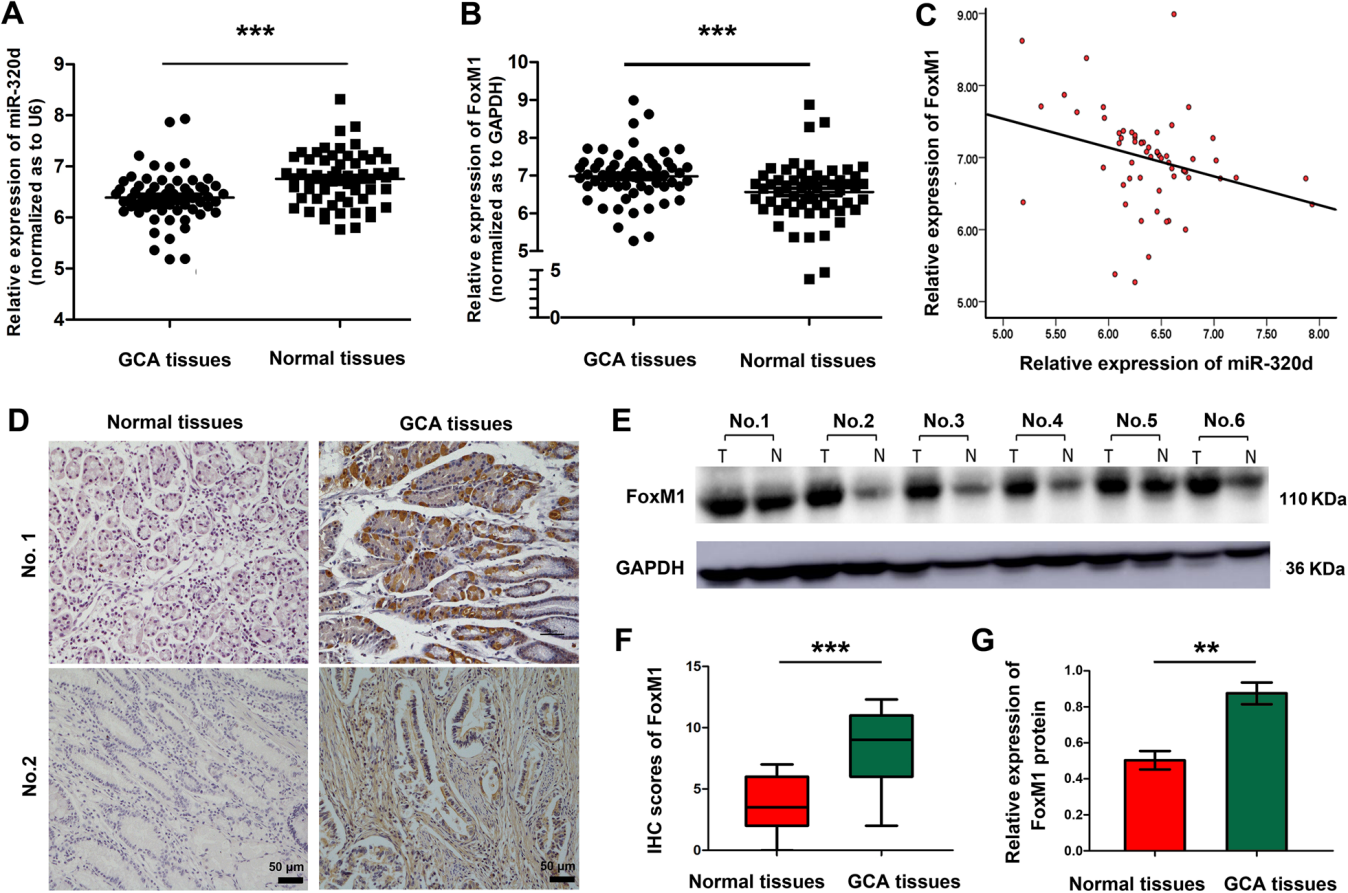
The miR-320d and FoxM1 expression level in GCA tissues and the adjacent normal tissues. RT-qPCR analysis of miR-320d expression level (**a**) and FoxM1 mRNA level (**b**) in 60 GCA tissues and the adjacent non-cancerous tissues. **c** Correlation between miR-320d expression and FoxM1 mRNA expression in 60 GCA tissues (*r* = − 2.94, *P* = 0.023). Representative IHC images (**d**) and western-blot analysis (**e**) of FoxM1 protein in GCA and normal tissues. **f** IHC scores of FoxM1 protein in GCA and normal tissues evaluated by the independent pathologists (n = 4). **g** Quantification of FoxM1 level by western-blot (n = 6). GAPDH was normalized as 100%. The * represents significant difference from GCA tissues to the normal tissues (**: *P* < 0.01; ***: *P* < 0.001). Data are shown as mean ± SD

60 fresh GCA tissues and adjacent normal tissues were collected to detect the expression of miR-320d and FoxM1. The clinicopathological characteristics of GCA patients were summarized in Table 1. Results from RT-qPCR assay showed that the miR-320d expression level was significantly decreased in GCA tissues when compared to the adjacent normal tissues (P < 0.001, Fig. 1a). However, the miR-320d expression level didn’t show a significant correlation with other characteristics of patients, such as age, sex, the stomach disease, pathological grade of GCA, et al. (Table 2). On the contrary, the FoxM1 mRNA expression level was significantly higher in tumor tissues than in the adjacent normal tissues (P < 0.001, Fig. 1b). The Spearman’s rank correlation analysis further clarified that the FoxM1 mRNA expression and miR-320d expression in GCA tissues are negatively correlated (r = − 2.94, *P* = 0.023, Fig. 1c). In addition, immunohistochemical (IHC) staining (Figure d and f) and western blot (Figure e and g) results also confirmed that FoxM1 protein was significantly enhanced in GCA tissues when compared with that in normal tissues. Among these 60 patients, the patients (n = 50) with positive lymph node metastases had higher FoxM1 mRNA expression when compared with patients with negative lymph node metastases (n = 10), instead of age, sex, and pathological grade of GCA (Table 2).

**Table 2:** Correlation between miR-320d or FoxM1 expression with clinicopathological features in 60 GCA patients

Characteristics	No. of patients	Relative miR-320d expression	*P*	Relative FoxM1 expression	*P*
Age (years)					
< 60	19	6.458 ± 0.272	0.106	7.151 ± 0.360	0.133
≥ 60	41	6.392 ± 0.146		6.924 ± 0.553	
Male/female					
Male	44	6.424 ± 0.201	0.225	7.115 ± 0.628	0.151
Female	16	6.405 ± 0.105		6.937 ± 0.563	
The stomach diseases					
No	18	6.438 ± 0.216	0.175	7.026 ± 0.660	0.207
Yes	42	6.398 ± 0.281		6.973 ± 0.478	
Family history of cancer					
No	27	6.419 ± 0.136	0.353	7.091 ± 0.512	0.116
Yes	33	6.409 ± 0.237		6.957 ± 0.730	
Pathological grade					
Low differentiated	19	6.607 ± 0.116	0.054	7.103 ± 0.621	0.051
Middle and high differentiated	41	6.492 ± 0.135		6.811 ± 0.672	
Clinical stage					
Low grades I–II	12	6.420 ± 0.127	0.307	7.020 ± 0.473	0.201
High grades III–IV	48	6.405 ± 0.138		6.977 ± 0.337	
Lymph node metastasis					
Negative	10	6.501 ± 0.217	0.057	6.863 ± 0.445	*0.044*
Positive	50	6.399 ± 0.138		7.162 ± 0.706	

*P* value < 0.05 is marked in italic, which means there is a significant difference between the two groups

*GCA* Gastric cardia adenocarcinoma

### Downregulation of miR-320d or overexpression of FoxM1 predicts poor prognosis of GCA

**Fig. 2:**
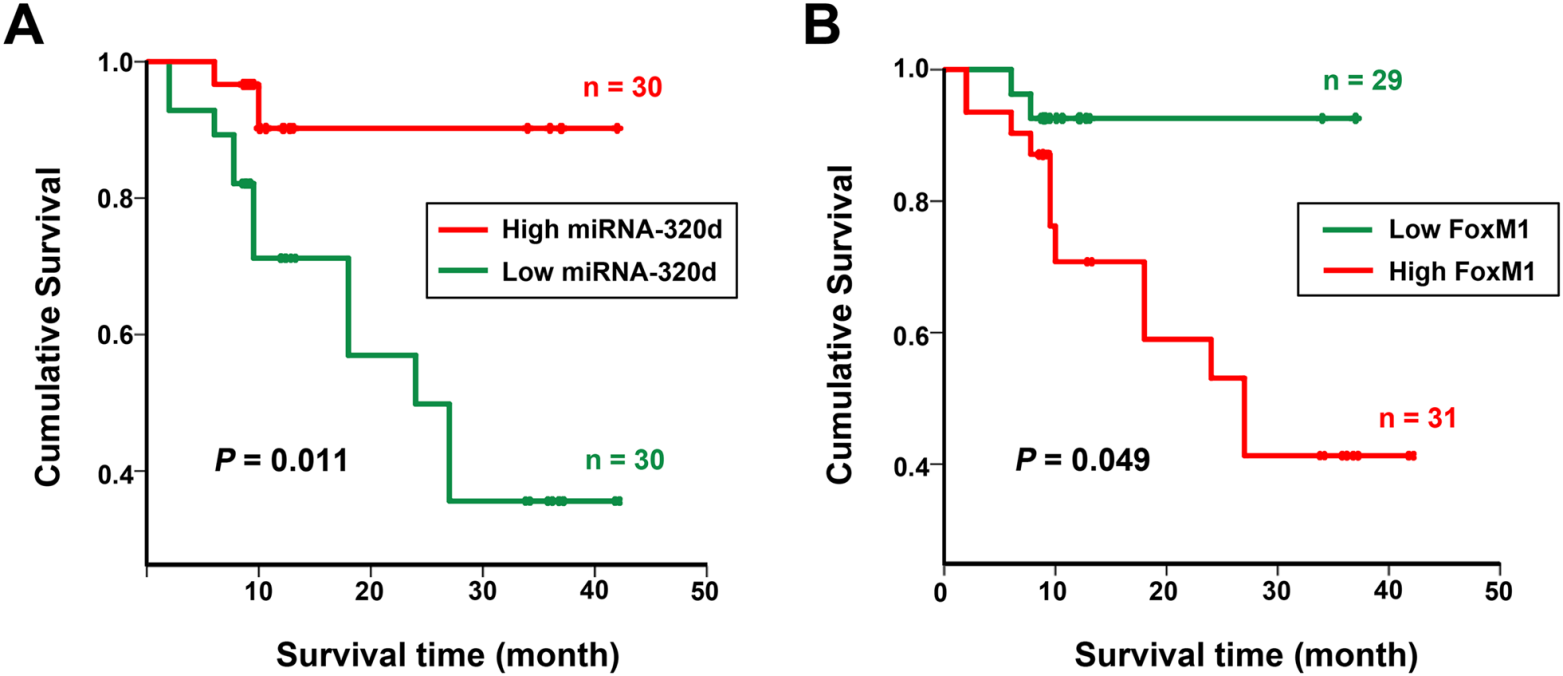
The effects of miR-320d and FoxM1 on overall survival of GCA patients. **a** Correlation between miR-320d expression and survival time. **b** Correlation between FoxM1 mRNA expression and survival time

Next we investigated the effects of miR-320d and FoxM1 on clinical outcome of GCA patients. According to the median level of miR-320d expression in tumor tissues, the total of 60 GCA patients were divided into 2 groups including high miR-320d expression group (n = 30) and low miR-320d expression group (n = 30). Similarly, high FoxM1 expression was found in 31 patients while low FoxM1 expression was involved in 29 patients (Fig. 2). The follow-up time was included from 15 to 44 months, and 14 patients died due to the recurrence and metastasis during this period (Table 1). In this study, Kaplan–Meier method and log-rank test were performed to evaluate the effects of miR-320d and FoxM1 on overall survival of the GCA patients. The results showed the overall survival time of patients with high expression of miR-320d was significantly longer than those with low expression miR-320d (median survival time: 44 months vs 24 months) (*P* = 0.011, Fig. 2a), implying poor prognosis of GCA correlated with low expression of miR-320d. The median survival time of GCA patients with high or low FoxM1 expression was 27 months or 44 months, respectively (*P* = 0.049, Fig. 2b), which demonstrated that high expression of FoxM1 is associated with poor prognosis of GCA.

### MiR-320d is negatively correlated with FoxM1 in GCA cells

**Fig. 3:**
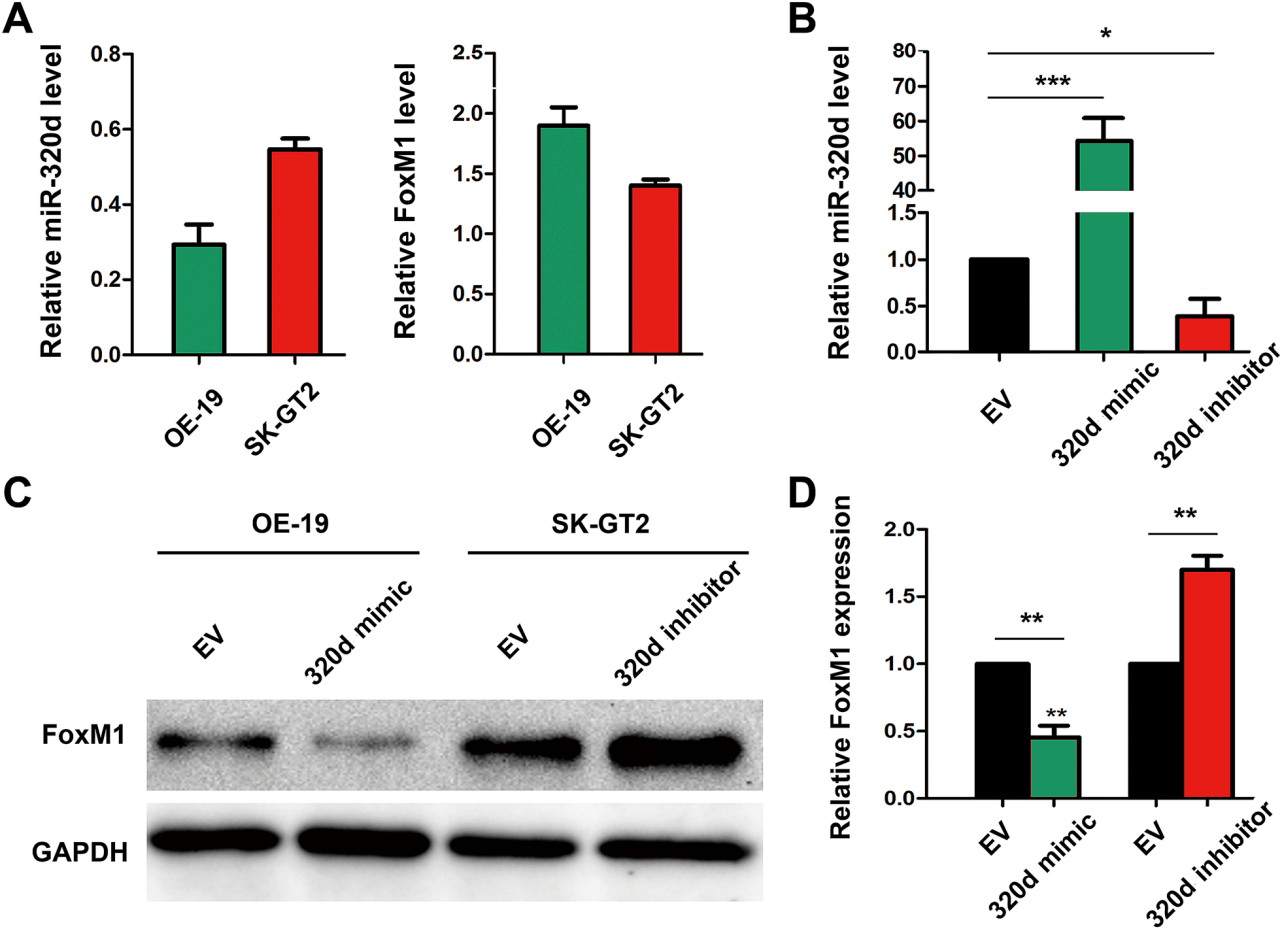
The regulatory relationship of miR-320d and FoxM1 in GCA cell lines. **a** RT-qPCR analysis of miR-320d and FoxM1 expression levels in GCA cell SK-GT2 and OE-19. **b** The expression of miR-320d in OE-19 cell that transfected with miR-320d mimics or in SK-GT2 cell with miR-320d inhibitor transfection. Western blot images (**c**) or quantification (**d**) of FoxM1 expression in GCA cells with or without transfection. The empty vector (EV) was set as control, and GAPDH was normalized as 100%. The * represents significant difference from miR-320d vector-transfected cells to EV-transfected cells (*: *P* < 0.05; **: *P* < 0.01; ***: *P* < 0.001). Data are shown as mean ± SD

As miR-320d level was negatively correlated with FoxM1 expression in GCA tissues, we further confirmed these correlation by in vitro experiments. GCA cell lines SK-GT2 and OE-19 were chosen as cell models as SK-GT2 cell is overexpressed miR-320d while OE-19 cell has low expression level of miR-320d (Fig. 3a, left). As expected, the FoxM1 expression level in SK-GT2 cell is lower than that in OE-19 cells (Fig. 3a, right). After transfecting SK-GT2 cell with miR-320d inhibitor, or OE-19 cell with miR-320d mimics (efficacy of transfection was shown in Additional file 1: Fig. S1), the expression levels of miR-320d was significantly downregulated in SK-GT2 cell (*P* = 0.03), and upregulated over 50-folds in OE-19 cell (*P* < 0.0001) (Fig. 3b). Moreover, the western blot results showed that FoxM1 protein expression level in OE-19 cell was significantly decreased by miR-320d mimics transfection while the FoxM1 level in SK-GT2 cell was significantly enhanced after miR-320d inhibitors transfection (Fig. 3c, d). These in vitro data corroborated GCA tissue data in Fig. 1.

### MiR-320d suppresses while FoxM1 promotes GCA cell proliferation and migration

**Fig. 4:**
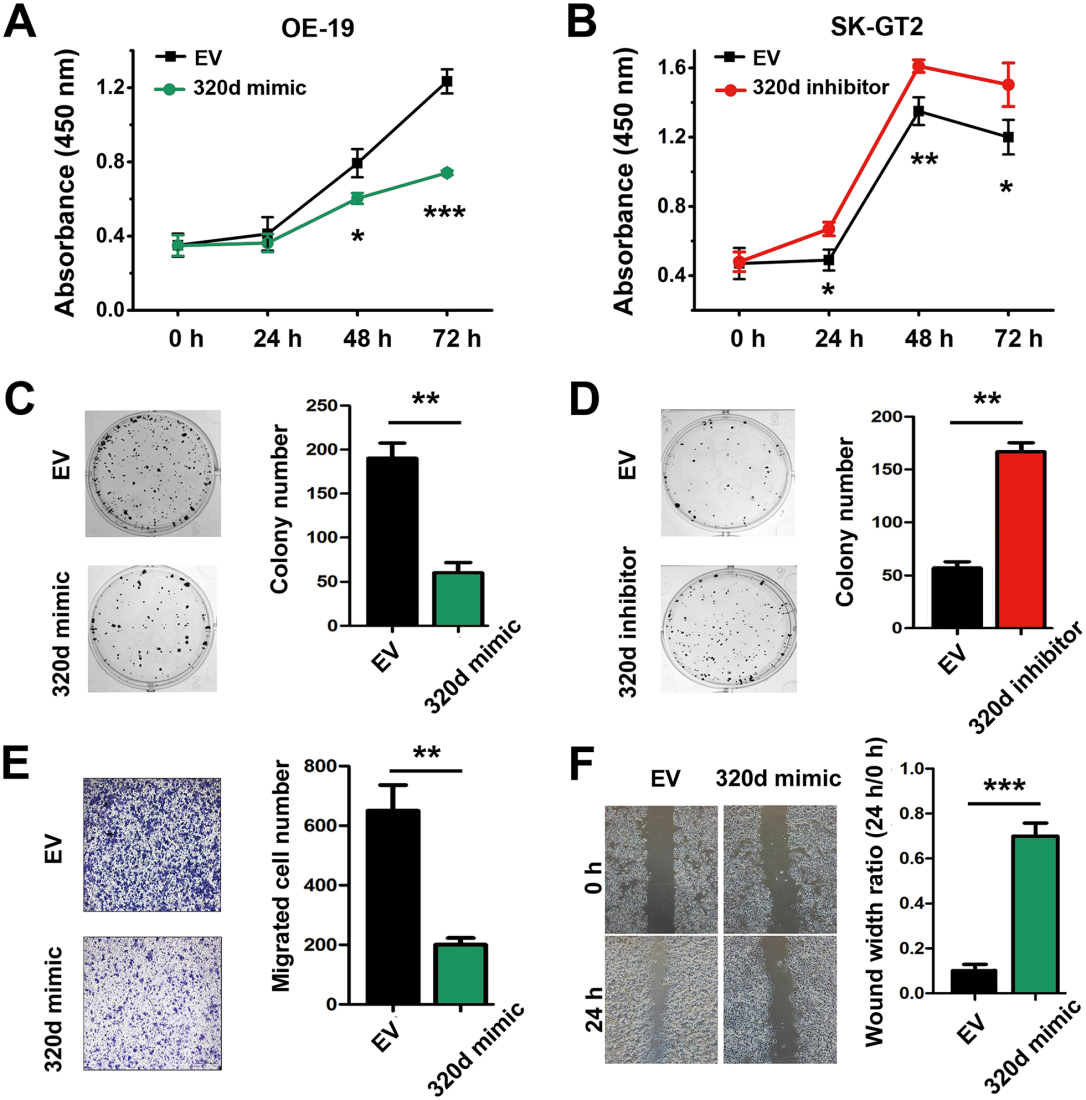
Effect of miR-320d on cell proliferation and invasion abilities in GCA cell lines. Up-regulated miR-320d inhibited the proliferation of OE-19 cells by CCK-8 assay (**a**) and colony formation assay (**c**). Down-regulated miR-320d promoted the proliferation of SK-GT2 cells by CCK-8 assay (**b**) and colony formation assay (**d**). Up-regulated miR-320d inhibited the invasion ability of OE-19 cell by transwell assay (**e**) and wound healing assay (**f**). The * represents significant difference from miR-320d vector-transfected cells to EV-transfected cells (*: *P* < 0.05; **: *P* < 0.01; ***: *P* < 0.001). Data are shown as mean ± SD

As miR-320d expression was significantly associated with clinical outcomes of GCA patients, we hypothesized miR-320d may affect GCA cell biological process. Therefore, CCK-8 assay was performed to evaluate the effect of miR-320d on GCA cell proliferation. The results showed that OE-19 cell growth rate was significantly suppressed after transfection of miR-320d mimics (Fig. 4a), while SK-GT2 cell growth rate was significantly increased after miR-320d inhibitor transfection (Fig. 4b). Moreover, mimic of miR-320d expression in OE-19 cell could significantly decrease the cell colony number (Fig. 4c), while inhibiting miR-320d expression in SK-GT2 cell promoted the cell colony number (Fig. 4d). Transwell assay and wound healing assay were used to verify the effect of miR-320d on cell migration in GCA cells. Up-regulation of miR-320d in OE-19 cells reduced the number of cell invading through the chamber inserts (Fig. 4e) and migrating ability (Fig. 4f). However, due to the weak invasive ability of SK-GT2 cell that has been spotted by others [22], we didn’t observe the obvious enhancement of cell invasion ability after down-regulation of miR-320d in SK-GT2 cells (Additional file 1: Fig. S2). The results above indicated that dysregulation of miR-320d can strongly affect GCA cell biological abilities, such as cell proliferation and migration, and thus influencing tumor progress and prognosis of patients with GCA.

### MiR-320d suppresses the GCA tumor growth in xenografted mice

**Fig. 5:**
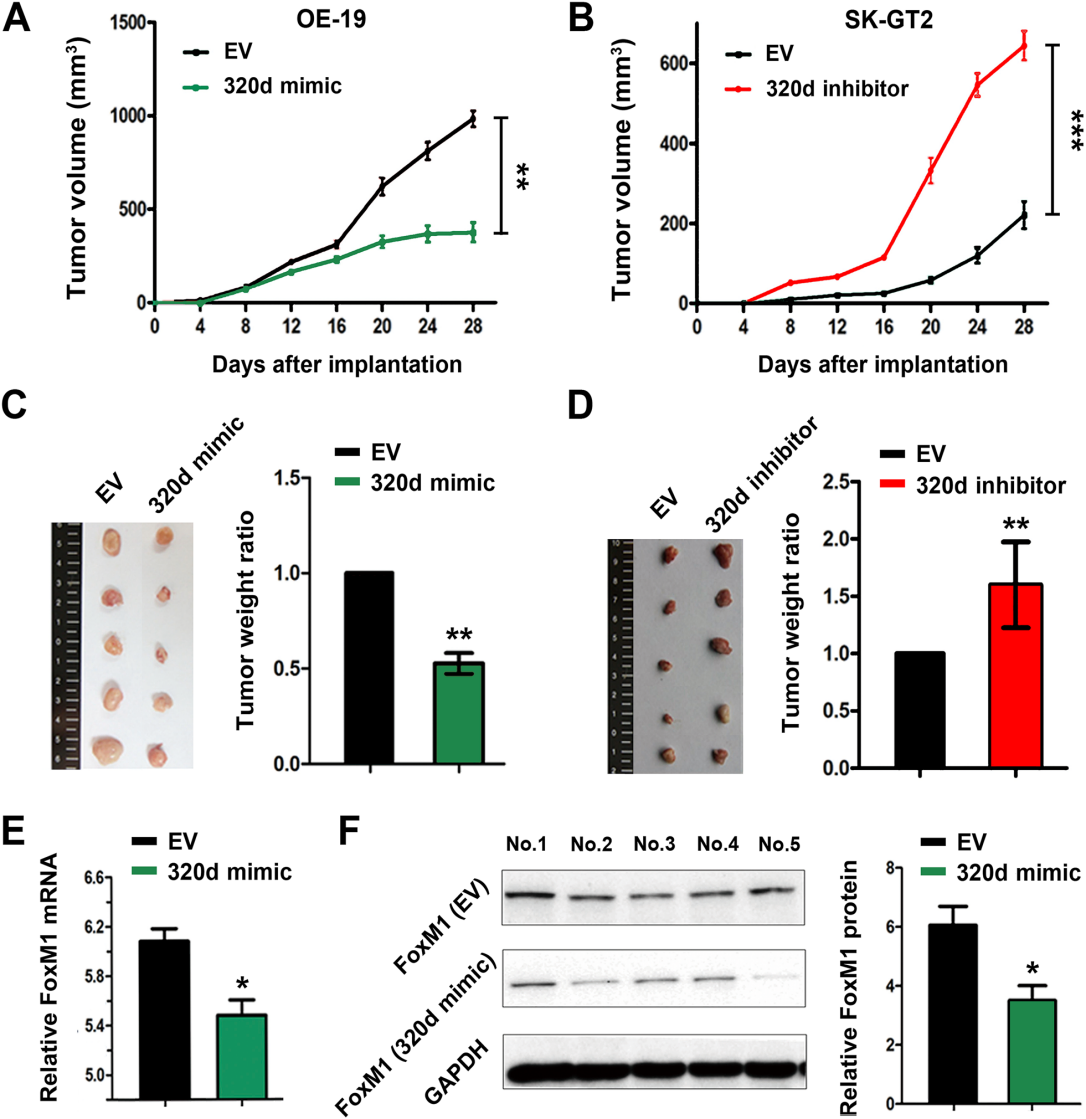
miR-320d/FoxM1 effect on the GCA tumor-bearing mice. Nude mice were subcutaneously injected with GCA cells. **a** Tumor volume of the mice implanted with EV-transfected or 320d mimics-transfected OE-19 cells during 4-weeks measurement. **b** Tumor volume of the mice implanted with EV-transfected or 320d-inhibitor transfected SK-GT2 cells. At the 4-weeks post-implantation, the mice were sacrificed and tumors were collected. **c** Tumor size in EV-transfected group or miR-320d mimics-transfected group. **d** Tumor size in EV-transfected group or 320d-inhibitor transfected group. **e** RT-qPCR analysis of FoxM1 mRNA in EV-transfected tumors or miR-320d mimics-transfected tumors. **f** Western-blot detected the expression of FoxM1 protein in EV-transfected tumors or miR-320d mimics-transfected tumors. The * represents significant difference from miR-320d vector-transfected tumors to EV-transfected tumors (*: *P* < 0.05; **: *P* < 0.01; ***: *P* < 0.001). Data are shown as mean ± SD

Encouraged by the in vitro results, we evaluated our findings into an in vivo model. Nude mice were subcutaneously injected EV-transfected OE-19, miR-320d mimics-transfected OE-19, EV-transfected SK-GT2, or miR-320d inhibitor-transfected SK-GT2 cells, and tumor size was measured every 4 days. As shown in Fig. 5, miR-320d mimics significantly inhibited OE-19 tumor growth (Fig. 5a), while down-regulation of miR-320d significantly promoted SK-GT2 tumor growth (Fig. 5b), which were well consistent with above in vitro data. After 4 weeks of tumor growth, mice were sacrificed and tumor were harvested for ex vivo analysis. Collected data showed that the average tumor volumes were 943.8 mm^3^ and 306 mm^3^ in EV-transfected OE-19 group and miR-320d mimics-transfected OE-19 group (Fig. 5c), respectively, and 219 mm^3^ and 631.4 mm^3^ in EV-transfected SK-GT2 group and miR-320d inhibitor-transfected SK-GT2 group, respectively (Fig. 5d). Compared to EV-transfected group, the tumor weight reduced by 50.57% in the miR-320d mimics-transfected group (Fig. 5c), and increased by 51.31% in the miR-320d inhibitor-transfected group (Fig. 5d). RT-qPCR and western blot results showed that miR-320d significantly decreased the expression of FoxM1 both in mRNA level and protein level (Fig. 5e, f). Meanwhile, miR-320d inhibitor increased the expression of FoxM1 mRNA in SK-GT2 tumors (Additional file 1: Fig. S3). As confirmed by IHC staining of FoxM1 protein, miR-320d mimics significantly decreased the expression of FoxM1 while miR-320d inhibitor increased FoxM1 expression (Additional file 1: Fig. S4). The data above strongly demonstrate that upregulation or downregulation of miR-320d in GCA tumors can significantly influence the FoxM1 level, thus affect tumor growth in tumor-bearing mice.

### miR-320d may directly target the 3′-UTR of FoxM1 in GCA cells

**Fig. 6:**
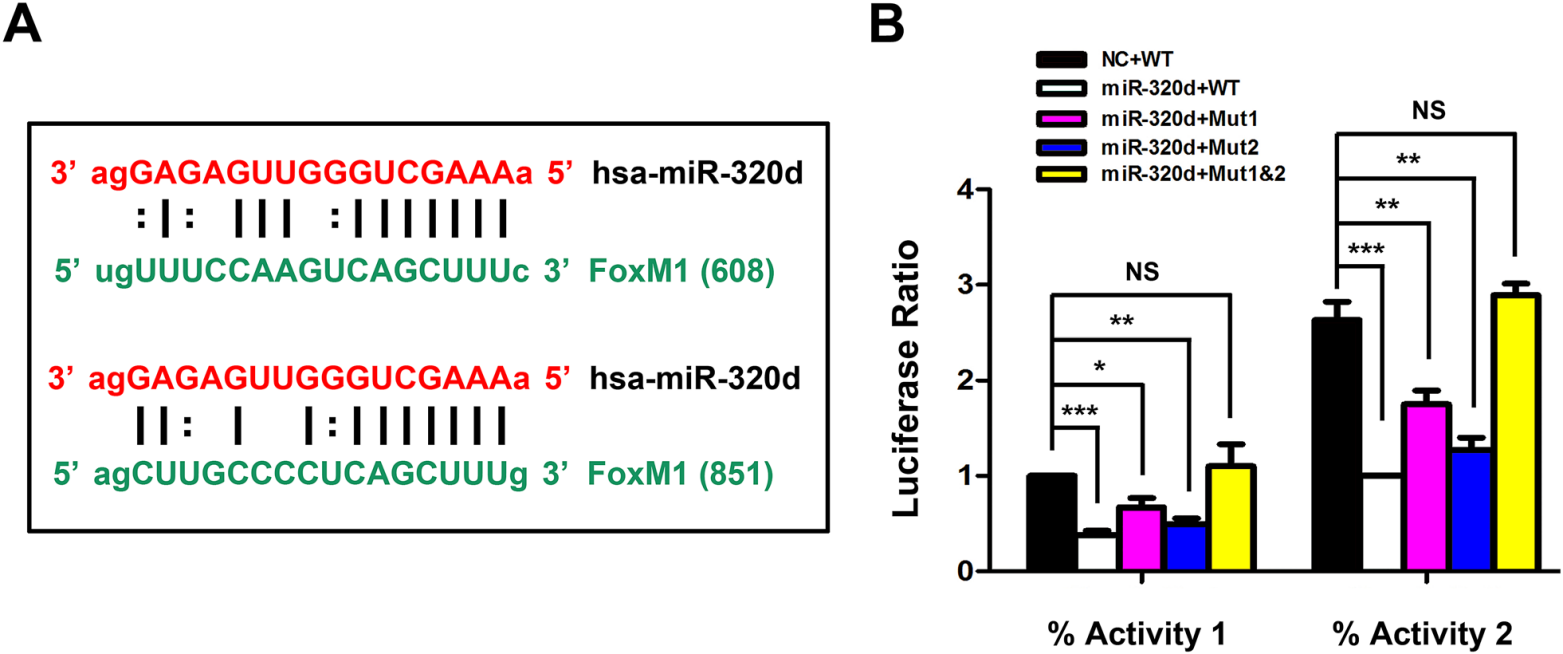
Confirmation of miR-320d/FoxM1 3´-UTR binding sites by dual luciferase activity reporter assay. **a** Two possible binding sites between miR-320d and FoxM1 3´-UTR were obtained by screening through TargetScan website. **b** Measurements of two luciferase activities in OE-19 cells after miR-320 mimics and 3´-UTR of FoxM1 plasmid co-transfections. NC was set as negative plasmid for miR-320 mimics. WT was the wild type of 3´-UTR of FoxM1. Mut1 was mutation of binding site 1 of 3´-UTR of FoxM1, while Mut2 was mutation of binding site 2. Mut1&2 was mutation of both two binding sites of 3´-UTR of FoxM1. The * represents significant difference from miR-320d mimics-transfected cells to EV-transfected cells (negative control) (*: *P* < 0.05; **: *P* < 0.01; ***: *P* < 0.001). NS represents no significance. Data are shown as mean ± SD

To finally identify whether the binding sites of 3′-UTR of the FoxM1 mRNA, which was obtained through microRNA website (TargetScan), are truly responsible for miR-320d/FoxM1 targeting, we conducted dual luciferase activity reporter assay for verification. The two sequences of 3′-UTR of FoxM1 for miR-320d binding were shown in Fig. 6a, one with a conserved sequence (UGUUUCCAAGUCAGCUUUC), while other one with a less conserved sequence (AGCUUGCCCCUCAGCUUUG). We constructed two mutants by mutating each site of 3′-UTR, respectively, and then transfected OE-19 cells with EV plasmid or miR-320d mimics plasmid, combined with wild type, or mutant 1 plasmid, or mutant 2 plasmid, or both mutant 1 and mutant 2 plasmid of FoxM1 3′-UTR. After co-transfection, the cells were collected for dual luciferase activity analysis. As shown in Fig. 6b (white column), manual overexpression of miR-320d significantly reduced the both luciferase activities (activity 1 and activity 2) in cell that co-transfected with wild type of FoxM1 3′-UTR, which suggesting that miR-320d could suppress FoxM1 expression level in GCA cells. When binding site 1 or site 2 was mutated, efficacy of miR-320d in suppressing FoxM1 was decreased, and even completely eliminated by mutation both site 1 and site 2 (Fig. 6b, yellow column). It should be noted that mutation of binding site 1 (Fig. 6b, purple column) could be more effective in reducing efficacy of miR-320d in suppressing FoxM1 than mutation of binding site 2 (Fig. 6b, blue column), which was ascribed to the more conserved sequence maintained in binding site 1. These results suggested that miR-320d can directly bind to the 3′-UTR of FoxM1 and thus regulate cell function.

## Discussion

MicroRNAs (miRNAs) are emerging as regulators of the gene expression network through RNA induced silencing complex mode of action, and accumulated evidence has shown that miRNAs have vital roles in most of cell biological processes. Though numerous studies have been focused on miRNAs, the functions of most miRNAs remain poorly understood. MiRNA-320d (miR-320d) is a member of miRNA-320 family, and acts as a tumor suppressor but has a very low expression level in several human malignant tumors [14–16]. However, very few researches have focused on this miRNA effects on gastric cardia adenocarcinoma (GCA). In the present study, we analyzed 60 GCA tissues and firstly reported that miR-320d was significantly down-regulated in GCA tissues. After follow-up those 60 GCA patients, the statistical analysis concluded that the miR-320d could predict the prognosis of GCA, that is the lower miR-320d has a poorer prognosis outcome in GCA patients. By examining effects of miRNA-320d on cell function we found that miR-320d can inhibit proliferation and invasion of GCA cells in vitro, suggesting that miRNA-320d may functionalize as a tumor suppressor by affecting tumor growth as well as metastasis.

Although recent studies have reported the functions of miR-320d in several cancer types, such as colorectal cancer [15], renal cell carcinoma [16], glioma [23], et al., less studies are focusing on the potential targeted genes of miR-320d. As the classical function of microRNA is silencing targeted genes by completely or incompletely pairing to the 3′-UTR of the specific genes, we screened the miR-320d targeted genes through the TargetScan website and other microRNA databases, and eventually found that FoxM1 contains two mRNA 3′-UTR binding sites for miR-320d. The context scores of these sites are very high. Additionally, through the TCGA database, it revealed that FoxM1 is overexpressed in most cancer types, and the expression level is also associated with disease prognosis [17–21]. Therefore, in the present study, we associated FoxM1 with miR-320 in human GCA tissues, GCA cell lines and GCA-tumor-bearing mice, and found a negative correlation between miR-320d expression and FoxM1 expression, that is down-regulation of miR-320d could lead to increase FoxM1 expression both in mRNA level and protein level, which promote tumor progression and resulting in poor clinical prognosis.

FoxM1 is a nuclear protein that controls multiple mitosis-required proteins and enzymes and affects cell growth by regulating cell cycle different phases [24]. Therefore, most cancer cells produce high level of FoxM1 to maintain their rapid growth [25]. Up to now, accumulated studies have revealed the downstream pathways that regulated by FoxM1. Ning et al. [18] demonstrated that FoxM1 can trigger PI3K/AKT, MEK/ERK, NF-κB signaling pathways, which accounts for cell migration and proliferation. Other research groups showed that downregulation of FoxM1 could affect intracellular MARK/p38, EGFR, COX-2 signaling pathways, thus regulating cell behaviors [24, 26, 27]. Our presented data demonstrated that up-regulation or down-regulation of miRNA-320d can significantly affect FoxM1 level in GCA cells, and the abilities of GCA cell proliferation, migration and invasion were influenced afterwards. Therefore, it is reasonable to conclude that the molecular mechanism of those cell behaviors could be via activating or inhibiting FoxM1-associated signaling pathways. On the other hand, in view of the evidences that overexpression of FoxM1 is positively associated with poor prognosis of several cancers, downregulation of FoxM1 by silencing FoxM1 mRNA currently has become one attractive strategy in cancer therapies [26, 27]. Miyashita et al. [17] investigated FoxM1 biological function in melanoma cells and demonstrated that FoxM1 could be a new promising target for treatment of melanoma. In this study, we up-regulated or down-regulated the expression of miRNA-320d in subcutaneously transplanted GCA tumors, and found that the growth rate of GCA tumor was significantly reduced or increased, respectively. Most importantly, the FoxM1 level was negatively associated with miR-320d level in GCA xenografted tumors. These further implied the molecular mechanism of miR-320d for tumor progression may be through regulating FoxM1 in GCA tumors. The proved relationship of miR-320d/FoxM1 in regulating the occurrence and development of GCA can show a promising strategy for GCA treatment by regulating miR-320d/FoxM1.

In summary, the observed remarkable negative correlation between miR-320d and FoxM1 in human GCA specimens, GCA cell lines, and GCA tumor-bearing mice, are achieved. The possible mechanism of them is that FoxM1 has two binding sites in 3´-UTR for miR-320d targeting. Given that FoxM1 is one of target genes for miR-320d, we speculate that FoxM1-mediated signaling pathways can be responsible for tumorigenesis, metastasis, and drug-resistance in malignant tumor development following dysregulation of miR-320d. Therefore, these findings demonstrated that miR-320d may potentially serve as a biomarker for cancer prognosis, and could be a novel target for gene therapy in conquering GCA disease.

## Supplementary information

**Additional file 1: Figure S1.** Efficiency of lentivirus transfection into GCA cell lines. OE-19 cells were transfected lentivirus with GFP, while SK-GT2 cells were transfected with e-cherry. At 48 h post-transfection, cells were fluorescently imaged by microscope. Compared with bright field images and fluorescence images, it could conclude that GFP-transfected OE-19 cells were more than 90%, and e-cherry-transfected SK-GT2 cells was more than 90%, which showed high efficacy of lentivirus transfection. **Figure S2.** Effect of miR-320d on cell invasion ability in SK-GT2 cell. Wound healing assay was performed to demonstrate the cell invasion ability after down-regulation of miR-320d in SK-GT2 cell. (A) Representative bright-field microscope images of SK-GT2 cell showing wound healing status after down-regulation of miR-320d at 0 and 24 h. (B) Quantification of SK-GT2 cell invasion ability rates after down-regulation of miR-320d. The NS represents that there is no significant difference between these two groups. **Figure S3.** RT-qPCR quantification of FoxM1 mRNA in EV-transfected SK-GT2 tumors and 320d-inhibitor-transfected SK-GT2 tumors. Nude mice were subcutaneously injected with 1x10^6^ EV-transfected SK-GT2 cells or 1 × 10^6^ 320d-inhibitor-transfected SK-GT2 cells. After 4 weeks later, the tumors were harvested for FoxM1 mRNA quantification. The FoxM1 mRNA level was increased in 320d-inhibitor transfected SK-GT2 tumor. The * represents significant difference from miR-320d inhibitor-transfected tumors to EV-transfected tumors (***: *P*  < 0.001). **Figure S4.** Immunohistochemical (IHC) analysis of FoxM1 protein expression in xenografted OE-19 tumors or SK-GT2 tumors. (A) IHC staining of FoxM1 antibody in xenografted tumor. Upregulation or downregulation of miRNA-320d can reduce or increase the expression level of FoxM1 protein in xenografted tumors. (B) The IHC scores of FoxM1 protein level in each group. The * represents significant difference from miR-320d vector-transfected tumors to EV-transfected tumors (**: *P*  < 0.01). **Table S1.** Primers for FoxM1 and GAPDH Gene Sequence. **Table S2.** Primers for FoxM1 3′-UTR (MT) clone and FoxM1 3′-UTR (WT) clone. **Table S3.** Group Settings for dual luciferase reporter assays (n = 5)

## Data Availability

The datasets used and/or analyzed during the current study are available from the corresponding author on reasonable request.
