# Supplementary material for: MicroRNA-320d regulates tumor growth and invasion by promoting FoxM1 and predicts poor outcome in gastric cardiac adenocarcinoma
Source: Cell Biosci. 2020 Jun 16;10:80. doi: 10.1186/s13578-020-00439-7 (PMC7298787; doi:10.1186/s13578-020-00439-7)
Supplement: Supplementary file 1 — Additional file 1: Figure S1. Efficiency of lentivirus transfection into GCA cell lines. OE-19 cells were transfected lentivirus with GFP, while SK-GT2 cells were transfected with e-cherry. At 48 h post-transfection, cells were fluorescently imaged by microscope. Compared with bright field images and fluorescence images, it could conclude that GFP-transfected OE-19 cells were more than 90%, and e-cherry-transfected SK-GT2 cells was more than 90%, which showed high efficacy of lentivirus transfection. Figure S2. Effect of miR-320d on cell invasion ability in SK-GT2 cell. Wound healing assay was performed to demonstrate the cell invasion ability after down-regulation of miR-320d in SK-GT2 cell. (A) Representative bright-field microscope images of SK-GT2 cell showing wound healing status after down-regulation of miR-320d at 0 and 24 h. (B) Quantification of SK-GT2 cell invasion ability rates after down-regulation of miR-320d. The NS represents that there is no significant difference between these two groups. Figure S3. RT-qPCR quantification of FoxM1 mRNA in EV-transfected SK-GT2 tumors and 320d-inhibitor-transfected SK-GT2 tumors. Nude mice were subcutaneously injected with 1x10^6^ EV-transfected SK-GT2 cells or 1 × 10^6^ 320d-inhibitor-transfected SK-GT2 cells. After 4 weeks later, the tumors were harvested for FoxM1 mRNA quantification. The FoxM1 mRNA level was increased in 320d-inhibitor transfected SK-GT2 tumor. The * represents significant difference from miR-320d inhibitor-transfected tumors to EV-transfected tumors (***: P < 0.001). Figure S4. Immunohistochemical (IHC) analysis of FoxM1 protein expression in xenografted OE-19 tumors or SK-GT2 tumors. (A) IHC staining of FoxM1 antibody in xenografted tumor. Upregulation or downregulation of miRNA-320d can reduce or increase the expression level of FoxM1 protein in xenografted tumors. (B) The IHC scores of FoxM1 protein level in each group. The * represents significant difference from miR-320d vector-transfected tumo [file 13578_2020_439_MOESM1_ESM.docx]

**MicroRNA-320d regulates tumor growth and invasion by promoting FoxM1 and predicts poor outcome in gastric cardiac adenocarcinoma**

**Xiaojie Chen****^1^,** **Shegan Gao^1,2,3,4^,** **Zhiwei Zhao^2^,** **Gaofeng Liang^1^,** **Jinyu Kong^2^, Xiaoshan Feng****^2,3,4^**^*^

^1^ Medical College, Henan University of Science and Technology, Luoyang, China

^2^ The First Affiliated Hospital, College of Clinical Medicine of Henan University of Science and Technology, Luoyang, China

^3^ Henan Key Laboratory of cancer Epigenetics, Henan University of Science and Technology, Luoyang, China

^4^ Cancer Institute, Henan University of Science and Technology, Luoyang, China

**
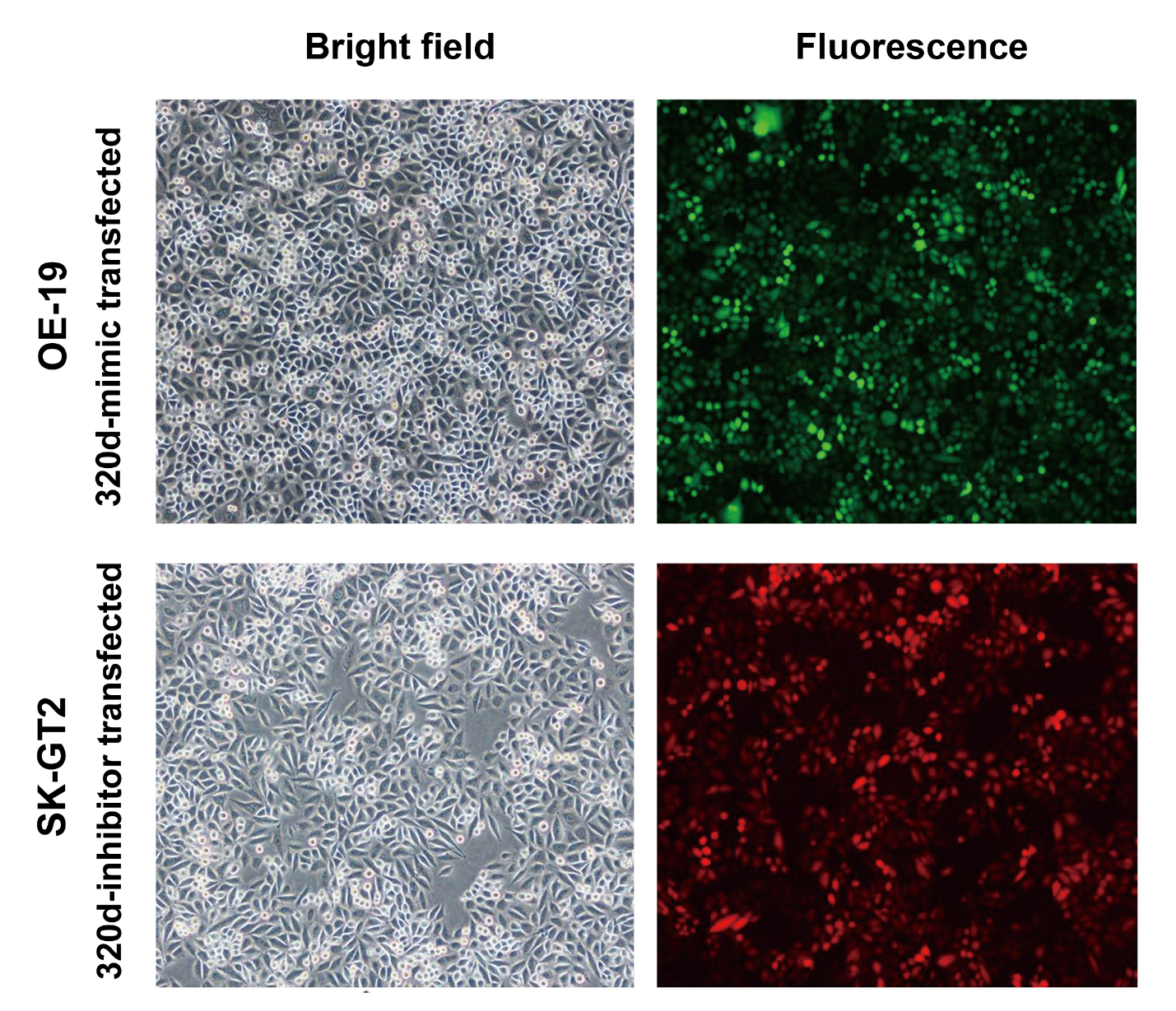
**

**Figure S1 Efficiency of lentivirus transfection into GCA cell lines.** OE-19 cells were transfected lentivirus with GFP, while SK-GT2 cells were transfected with e-cherry. At 48 h post-transfection, cells were fluorescently imaged by microscope. Compared with bright field images and fluorescence images, it could conclude that GFP-transfected OE-19 cells were more than 90%, and e-cherry-transfected SK-GT2 cells was more than 90%, which showed high efficacy of lentivirus transfection.


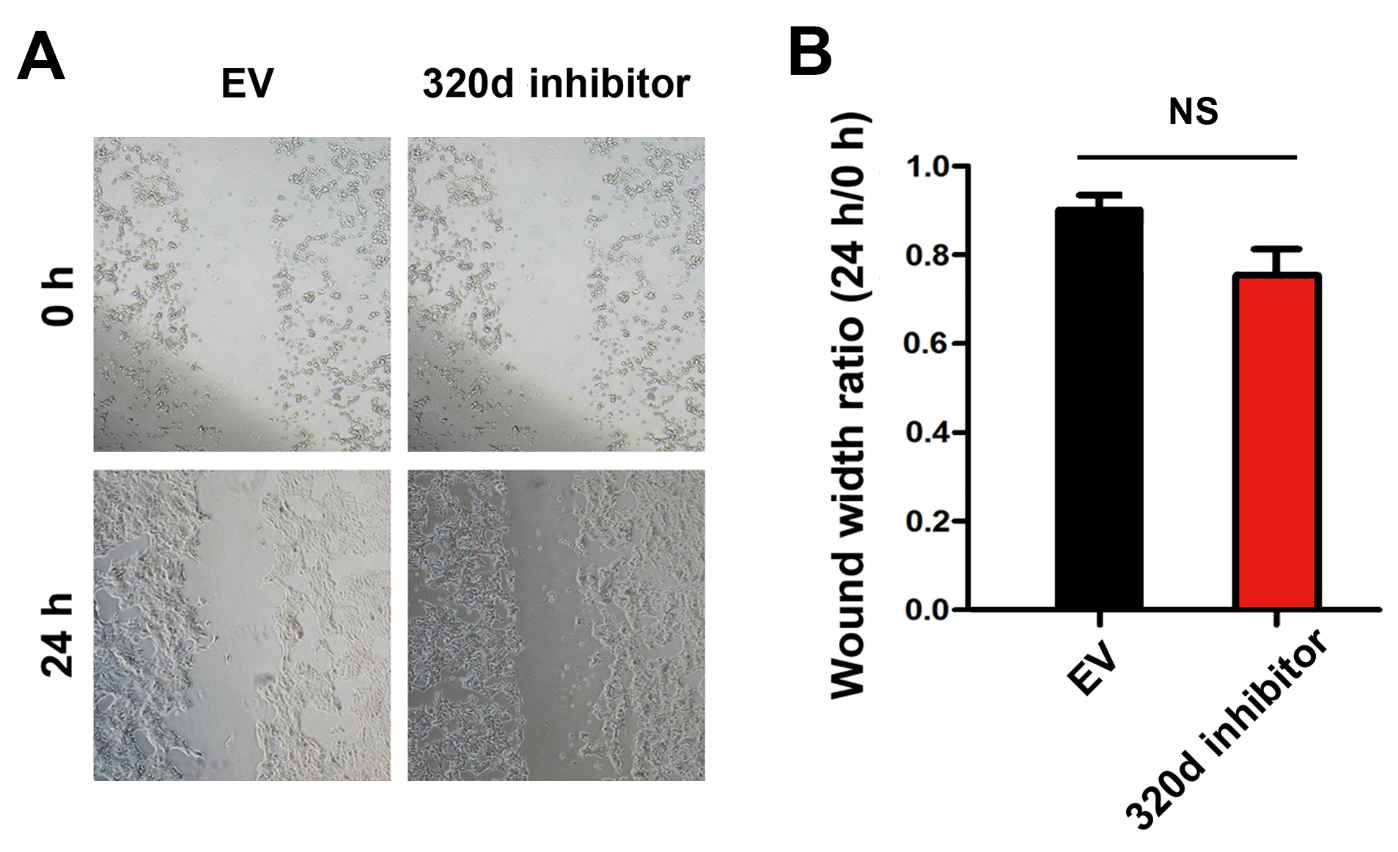


**Figure S2** **Effect of miR-320d on cell invasion ability in SK-GT2 cell.** Wound healing assay was performed to demonstrate the cell invasion ability after down-regulation of miR-320d in SK-GT2 cell. (A) Representative bright-field microscope images of SK-GT2 cell showing wound healing status after down-regulation of miR-320d at 0 and 24 h. (B) Quantification of SK-GT2 cell invasion ability rates after down-regulation of miR-320d. The NS represents that there is no significant difference between these two groups.


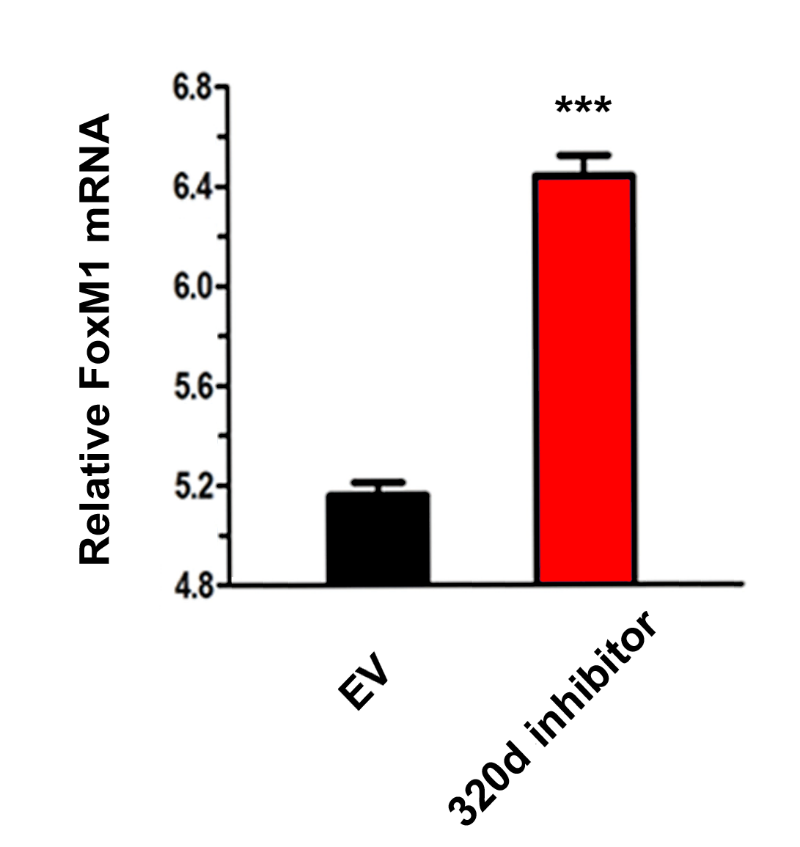


**Figure S3 RT-qPCR quantification of FoxM1 mRNA in EV-transfected SK-GT2 tumors and 320d-inhibitor-transfected SK-GT2 tumors.** Nude mice were subcutaneously injected with 1x10^6^ EV-transfected SK-GT2 cells or 1x10^6^ 320d-inhibitor-transfected SK-GT2 cells. After 4 weeks later, the tumors were harvested for FoxM1 mRNA quantification. The FoxM1 mRNA level was increased in 320d-inhibitor transfected SK-GT2 tumor. The * represents significant difference from miR-320d inhibitor-transfected tumors to EV-transfected tumors (***: *P* <0.001).


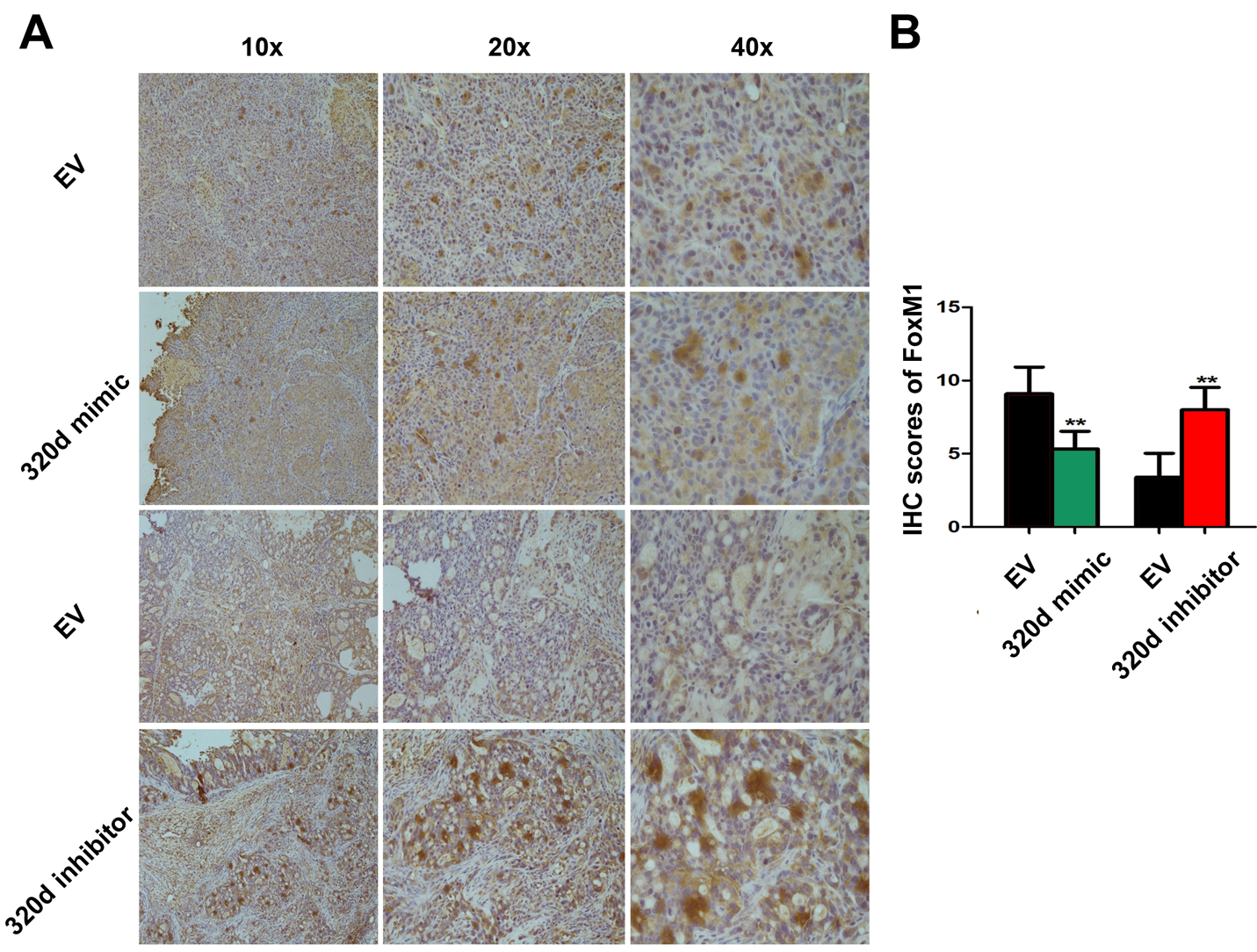


**Fig****ure S4 Immunohistochemical (IHC) analysis of FoxM1 protein expression in xenografted OE-19 tumors or SK-GT2 tumors.** (A) IHC staining of FoxM1 antibody in xenografted tumor. Upregulation or downregulation of miRNA-320d can reduce or increase the expression level of FoxM1 protein in xenografted tumors. (B) The IHC scores of FoxM1 protein level in each group. The * represents significant difference from miR-320d vector-transfected tumors to EV-transfected tumors (**: *P* <0.01).

**Table S1 Primers for FoxM1 and GAPDH Gene Sequence**

| **Gene** | **Sequence（5′-3′）** | **Cloned Length（bp）** |
| --- | --- | --- |
| **FoxM1-F** | AGCAGTCTCTTACCTTCC | 201 |
| **FoxM1-R** | CTGGCAGTCTCTGGATAA |  |
| **GAPDH-F** | CTCTGGTAAAGTGGATATTGT | 162 |
| **GAPDH-R** | GGTGGAATCATATTGGAACA |  |

**Table S2 Primers for FoxM1 3′-UTR (MT) clone and FoxM1 3′-UTR (WT) clone**

| **Gene** | **Sequence（5′-3′）** |
| --- | --- |
| **MutFoxM1-1F** | GCAGG TTTCGACACTTGGAAACACGGGGAGGTGGCAGGGA |
| **Mut FoxM1-1R** | CAAGTGTCGAAACCTGCAAGAAGAAATCCTGG |
| **MutFoxM1-2F** | CTTTGCTTTCGACAGGGGCAAGCTAAGGAAGCCAGG |
| **Mut FoxM1-2R** | GCCCCTGTCGAAAGCAAAGAGCCACCCTAGGCCC |
| **WT FoxM1-1F** | CCGCTCGAG AAGCAGCTGCCGCAGCTTGTC |
| **WT FoxM1-2R** | ATAAGAATGCGGCCGCTTTTAACATTTATTGAGAACTTTT |

MT: mutation type, WT: wild type.

**Table S3 Group Settings for dual luciferase reporter assays (n = 5)**

| Information  about clone | Wild type of **FoxM1** 3′-UTR clone | Mutation type of **FoxM1**  3′-UTR clone | | |
| --- | --- | --- | --- | --- |
|  | WT | Mut1 Mut2 Mut1&2 | | |
| miR-320d mimics | √ | √ | √ | √ |
| Empty vector | √ |  |  |  |

WT= wild type, Mut1 = mutate binding site 1, Mut2 = mutate binding site 2, Mut1&2 = mutate binding sites 1 and 2
